# Special Issue “Diagnosis and Management of Dry Eye Disease and Ocular Surface Inflammation”

**DOI:** 10.3390/medicina58060764

**Published:** 2022-06-05

**Authors:** Giuseppe Giannaccare, Antonio Di Zazzo

**Affiliations:** 1 Department of Ophthalmology, Magna Graecia University of Catanzaro, 88100 Catanzaro, Italy; 2 Department of Ophthalmology, University Campus Bio-Medico, 00128 Rome, Italy; antoniodizazzo@gmail.com

It is estimated that a wide proportion of the world’s population (5% to 50%) may suffer from dry eye disease to a various extent [1]. Since the prevalence is higher in older people, the global burden of the disease is projected to further increase in the next decades due to the ageing of populations. Nevertheless, there is still lack of reliable and validated biomarker(s) to diagnose the condition, monitor its course over time and guide clinical management. As such, the “real life” diagnosis is usually reached by combining multiple subjective and invasive tests performed at the slit-lamp, such as among others vital dye staining and tear film break-up time. However, these tests are observer-dependent and lack standardization, resulting in high interobserver and intraobserver variance [2,3]. Another important problem is the poor concordance between the objective signs and the symptoms experienced by patients, in particular in cases with neurosensory abnormalities [4].

To overcome these issues, recent non-invasive devices have been developed to measure various novel diagnostic parameters, such as non-invasive break-up time, tear meniscus height, infrared meibography, aberrometry, tear film interferometry, humidity, temperature and corneal nerves metrics [5,6,7,8,9,10]. All-in-one integrate devices combining multiple tests to obtain a comprehensive ocular surface evaluation are now available. These devices have the great advantage of providing automated results, thus improving the diagnostic repeatability and reproducibility. Moreover, given the complex and multifactorial nature of dry eye disease, the possibility to identify the ocular surface structure or the tear film layer mostly affected can allow for a targeted treatment.

In parallel with the development of novel diagnostic devices, new dry eye treatments have also been developed and introduced on the market. With regards to topical treatment, tear substitutes formulations have evolved into multiple-action combined formulas targeting different key mechanisms of the vicious dry eye cycle [11,12]. Soft corticosteroids, cyclosporine A, tacrolimus and lifitegrast have been indicated as possible useful and safe tools to control inflammation. Certain dietary constituents also seem to have positive effects on the ocular surface health, with omega-3 fatty acids having the strongest level of evidence in support of their use. The treatment of meibomian gland dysfunction has also progressively shifted from simple lid hygiene to novel in-office treatments based on devices administering therapeutic levels of heat and pressure/vibration [13], as well as intense pulse light [14] or low-level light therapy [15].

This collection of articles will provide a focused update on the most compelling diagnostic and therapeutic innovations for dry eye disease and ocular surface inflammation. As Guest Editors, we would like to thank the authors who will contribute to this Special Issue and the reviewers who will provide helpful suggestions to further improve the quality of the papers. We would also like to extend our gratitude to the team of the journal *Medicina* for their robust support in this project.

## Data Availability

Data sharing not applicable No new data were created or analyzed in this study. Data sharing is not applicable to this article.
